# Cross-cultural adaptation and psychometric properties of the Arabic version of the Central Sensitization Inventory in people with chronic musculoskeletal pain

**DOI:** 10.7717/peerj.18251

**Published:** 2024-10-08

**Authors:** Sarah E. Tamboosi, Hosam Alzahrani, Fahad H. Alshehri, Msaad Alzhrani, Yasir S. Alshehri

**Affiliations:** 1 Department of Physical Therapy, Al-khorma General Hospital, Taif, Saudi Arabia; 2 Department of Physical Therapy, College of Applied Medical Sciences, Taif University, Taif, Saudi Arabia; 3 Department of Physical Therapy and Health Rehabilitation, Majmaah University, Majmaah, Saudi Arabia; 4 Department of Physical Therapy, College of Medical Rehabilitation Sciences, Taibah University, Madinah, Saudi Arabia

**Keywords:** Central Sensitization Inventory, CSI, CSI-Ar, Pain, Cross-cultural adaptation, Psychometric validation

## Abstract

**Background:**

The Central Sensitization Inventory (CSI) is a patient-reported screening instrument that can be used to identify and assess central sensitization (CS)/Central Sensitization Syndrome (CSS)-related symptoms.

**Objective:**

The aim was to translate the CSI into Arabic (CSI-Ar) and to subsequently validate its psychometric properties.

**Design:**

Cross-sectional.

**Methods:**

The CSI was translated and cross-culturally adapted into Arabic, and validated following international standardized guidelines. This study included patients with chronic musculoskeletal pain (*n* = 264) and healthy control participants (*n* = 56). Patients completed the CSI-Ar, Pain Catastrophizing Scale (PCS), Depression, Anxiety, and Stress scale (DASS-21), Tampa Scale of Kinesiophobia (TSK), and 5-level EuroQol-5D (EQ-5D). Patients completed the CSI-Ar twice to assess test–retest reliability. To evaluate discriminative validity, healthy controls participants completed the CSI-Ar. Statistical analyses were conducted to test the internal consistency, reliability, and structural, construct and discriminant validity of CSI-Ar.

**Results:**

The CSI-Ar showed acceptable internal consistency (Cronbach’s alpha = 0.919) and excellent test–retest reliability (intraclass correlation coefficient = 0.874). The CSI-Ar scale had significant correlations (*P* < 0.001) with all PCS subscales and total score (Spearman’s rho = 0.459–0.563, *P* < 0.001), all DASS-21 subscales and total score (Spearman’s rho = 0.599–0.685, *P* < 0.001), the TSK (Spearman’s rho = 0.395, *P* < 0.001), and the EQ-5D (Spearman’s rho = −0.396, *P* < 0.001). The Mann-Whitney U-test showed a statistically significant difference between the patient group and the healthy control group (*P* < 0.001), with the healthy controls displaying a lower average CSI-Ar score (12.27 ± 11.50) when compared to the patient group (27.97 ± 16.08). Factor analysis indicated that the CSI-Ar is a unidimensional tool.

**Conclusion:**

The CSI-Ar is a reliable and valid screening tool that can be used to assess CS/CSS-related symptoms in Arabic-speaking people with chronic musculoskeletal pain.

## Introduction

Chronic musculoskeletal pain is a leading cause of disability worldwide (Vos et al., 2017). According to the Global Burden Disease (GBD) statistics, 1.75 billion individuals worldwide suffer from chronic musculoskeletal pain (Cieza et al., 2020). As a result of chronic musculoskeletal (MSK) pain, daily tasks and activities become more difficult, medicines are used more frequently, and there is a larger possibility of sick leave and disability pensions, which in turn have a negative impact on quality of life. Furthermore, it is a major public health concern, resulting in substantial costs for healthcare systems and disability insurance (Cimmino, Ferrone & Cutolo, 2011).

A number of studies have focused on the phenomenon of central nervous system hypersensitivity in chronic pain patients (Woolf, 2011). This phenomenon is known as central sensitization (CS). The CS is a neurophysiological disorder causes hyperexcitability of the central nervous system. According to Woolf, the CS is “operationally defined as an increase in neural signaling in the central nervous system that causes hypersensitivity to pain” (Woolf, 2011). The CS is indicated for a variety of chronic pain disorders, including fibromyalgia (Vierck, 2006), whiplash (Curatolo et al., 2001), low back pain (Roussel et al., 2013), and osteoarthritis (Lluch Girbés et al., 2013).

Yunus (2007) used the term “Central Sensitization Syndrome (CSS)” to describe a chronic disease in which CS appears to be a common cause. The author proposed renaming these disorders to (CSS) and introduced the idea that CS may be a common trait that causes similar overlapping symptoms in these syndromes. In addition to the absence of structural pathology, the majority of CSS share objectively a lower pain threshold and heightened pain sensitivity (Aaron & Buchwald, 2001), which is an important feature of the CS (Latremoliere & Woolf, 2009).

In the past few decades, a complete clinical patient-centered biopsychosocial assessment and therapy approach has been evacuated for this complex patient population (Nijs et al., 2014; Wijma et al., 2016). The ability to recognize when presenting symptoms are related to CS can help clinicians choose the most relevant and effective diagnostic and treatment approaches (Jull et al., 2007).

Mayer et al. (2012) developed the Central Sensitization Inventory (CSI) as a patient-reported screening tool that can be used to identify and quantify CS/CSS-related symptomology. The CSI concept is based on the CSSs paradigm, in which distinct diseases with different phenotypes share overlapping CS symptoms. Through a literature search, these symptoms were taken from the CSS paradigm and reduced into a single questionnaire (Yunus, 2007). The CSI is used to detect symptoms of CS, including widespread pain patterns, sleep disturbances, hypersensitivity to stimuli, and cognitive, digestive, and urological problems (Mayer et al., 2012). The CSI is a widely used tool that has demonstrated good reliability and validity in populations with chronic pain conditions (Mayer et al., 2012).

Recently, the CSI has received a lot of attention, and it has been translated, culturally adapted, and validated in numerous languages, including Brazilian Portuguese (Caumo et al., 2017), Dutch (Kregel et al., 2016), French (Pitance et al., 2016), Spanish (Cuesta-Vargas et al., 2016), Italian (Chiarotto et al., 2018), Serbian (Knezevic et al., 2018), and Japanese (Tanaka et al., 2017). While the CSI was also adapted and validated for use in Arabic-speaking populations, the study had several limitations (Madi et al., 2022). Firstly, it included participants with a wide range of chronic conditions, rather than focusing specifically on musculoskeletal pain, and it was conducted during the COVID-19 pandemic, which may have introduced variability that could affect the results. Secondly, the study did not assess potential floor and ceiling effects, which are crucial for understanding the responsiveness and interpretability of the CSI in this particular context. Thus, the aim of the current study was to translate and culturally adapt the CSI into the Arabic language (CSI-Ar), as well as to evaluate its test-retest reliability, construct validity, and discriminant validity in patients with chronic musculoskeletal pain disorders.

## Methods

### Design

The design of this study was cross-sectional. After gaining permission of the author of the original English version, the process of translating and validating the CSI into Arabic was started. This study consisted of two stages: First, translation and cross-cultural adaptation of the original CSI version into Arabic; Second, assessing the psychometric properties of the Arabic version of the CSI. The study protocol received approval from the Scientific Research Ethics Committee at Taif University (No. 44-003). Written informed consent has been obtained from all participants. This study adhered to the guidelines set forth by “The Strengthening the Reporting of Observational Studies in Epidemiology (STROBE)” and “COnsensus-based Standards for the selection of health Measurement INstruments (COSMIN)” guidelines (Von Elm et al., 2007; Gagnier et al., 2021).

### Translation and cross-cultural adaptation of the CSI

This stage followed the criteria for adapting self-report measures for cross-cultural adaptation and translation (Beaton et al., 2000):

**Step 1: forward translation.** The CSI was translated from English to Arabic with the goal of preserving the original questionnaire’s meaning. Two translations were completed by two translators who speak Arabic as their native language. The translators transferred the item to an appropriate cultural context when a concept had no equivalent in Arabic culture. A discussion between the two translators was held to determine the translational options for the most difficult terms. The translators then worked on combining the two translations into a single translation. None of the original items were excluded.

**Step 2: backward translation**. Two independent bilingual native English-speaking translators worked on the back translation from the Arabic version into English while taking into account social and cultural differences between the US and Arab. To reduce information bias and allow unexpected interpretations of questions in the translated questionnaire, the two translators were not aware about the topics being studied, and they did not have medical backgrounds.

**Step 3: expert committee.** A multilingual committee, which included our four translators, reviewed both forward and backward translations. To achieve conceptual equivalency, the group discussed various choices for items and responses, emphasizing meaning above literal translation.

**Step 4: testing the pre-final version**. The questionnaire was given to 50 patients who were randomly chosen from all patients at the participating facilities who met the inclusion and exclusion criteria to assess the clarity of items and responses of the Arabic CSI (CSI-Ar) and on how to revise them if necessary.

### Participants

This study included adults with chronic musculoskeletal pain for a minimum of 3 months, and who have enough knowledge of the Arabic language and sufficient physical and cognitive ability to participate. This study excluded those with a diagnosis of specific medical conditions that can negatively impact the central nervous system, including brain or spinal cord injury, cancer and/or neurological disease or injury. It also excluded participants with psychiatric disease with pain as the main symptom (for example somatoform disorders, severe depression), as well as participants whose rheumatological disease was in its initial or unstable phase, and/or participants with a primarily neuropathic pain component.

In addition, local contacts were used to recruit healthy control participants, without musculoskeletal pain, from the general population.

### Sample size calculations

The sample size was estimated following the methodology outlined by Boateng et al. (2018). It is suggested that a minimum of 10 participants per scale item is required, with an ideal ratio of 10:1. Because the CSI has 25 items, this study requires 250 participants. The aim was to recruit a minimum of 250 participants with chronic musculoskeletal pain and 50 healthy control participants.

### Administered questionnaires

#### Central Sensitization Inventory-Arabic version

The CSI is composed of two parts (A and B). Part A has 25 questions that assess the typical symptoms of CS/CSS. The severity of these symptoms is rated on a five-point Likert scale, from never to always (never = 0, rarely = 1, sometimes = 2, often = 3, always = 4). The single-item scores are added up to provide a total score ranging from 0 to 100. Part B of the questionnaire questions the patient about ten previously diagnosed disorders from their medical history, including seven common CSSs and three additional conditions associated with CS/CSS. Part B of the CSI is not assessed in the same way as Part A and is just intended to offer extra information (Mayer et al., 2012; Neblett, 2018). The authors have been granted permission from the copyright holders to translate and assess the psychometric properties of this instrument.

#### Depression anxiety stress scale

The Depression, Anxiety, and Stress Scale (DASS) is a tool developed for evaluating and assessing the depression, anxiety, and stress (Lovibond, 1995). The authors have obtained permission from the copyright holder to use this instrument. The DASS-21 is a new short version of DASS which includes three subscales, each containing seven items. Many studies have assessed the psychometric prosperities of this scale to determine its validity and reliability (Ali et al., 2017; Pezirkianidis et al., 2018; Yıldırım, Boysan & Kefeli, 2018; Jiang et al., 2020; Zanon et al., 2021).

#### Pain catastrophizing scale

The Pain Catastrophizing Scale (PCS) is used to quantify catastrophizing attitudes and beliefs about pain (Sullivan, Bishop & Pivik, 1995). It consists of 13 items, each of which is assessed on a five-point Likert scale, with a total score ranging from 0 to 52 (Meyer, Sprott & Mannion, 2008). The PCS has previously been translated, culturally adapted, and validated into Arabic (Terkawi et al., 2017). The authors have obtained permission from the copyright holder to use this instrument.

#### EuroQOL’s five-dimension questionnaire

The EQ-5D is a general, preference-based instrument that assesses three different aspects of quality of life (Rabin & De Charro, 2001). The first aspect is a descriptive system with a five-dimensional profile of respondents’ health state. The second aspect is a visual analog scale (VAS; 0–100) for rating one’s own health. The third aspect of the questionnaire is an index score that reflects the general public’s choice or utility for the measured health profile can be created. Previously, the tool was translated and validated into Arabic (Bekairy et al., 2018). The authors have obtained permission from the copyright holder to use this instrument.

#### Numeric pain rating scale

The Numeric Pain Rating Scale (NPRS) is an outcome measure that measures pain intensity in adults (Jensen & McFarland, 1993). Patients rate their pain using the 11-point numerical pain rating scale (NPRS), which has 11 different categories (Downie et al., 1978; Jensen, Turner & Romano, 1994; Price et al., 1994; Katz & Melzack, 1999). Furthermore, it has been demonstrated to have concurrent and predictive validity of pain intensity (Jensen, Turner & Romano, 1994; Price et al., 1994; Jensen et al., 1999; Katz & Melzack, 1999). The NPRS was previously translated into Arabic which was valid and reliable for measuring pain levels in patients with knee osteoarthritis (Alghadir, Anwer & Iqbal, 2016).

#### Tampa scale of kinesiophobia

The original Tampa Scale of Kinesiophobia (TSK) was first developed in 1991 and it measures the subjective level of kinesiophobia or fear of movement (Miller, Kori & Todd, 1991). It comprises of 17 questions, each of which asks about the severity of the symptoms and discomfort. The responses are scored on a four-point Likert scale, where “totally disagree” equals one point, “partially disagree” equals two points, “partially agree” equals three points, and “totally agree” equals four points. To calculate the overall score, the answers to questions 4, 8, 12, and 16 must be inverted. The potential score on the scale can be anywhere between 17 and 68 points, with a higher number indicating a higher level of kinesiophobia. The Arabic version of the TSK was also found to be reliable and valid (Yangui et al., 2017).

### Data collection

An online-based survey (Google Forms survey) was created for participants to fill out. The participants had the choice to fill out the questionnaires in the hospital or at home. Social media was used to invite eligible patients to participate in this study. Participants were kindly instructed to complete all the parts of the questionnaire.

### Statistical analysis

#### Descriptive analyses

All the analyses were conducted using the SPSS software (version 26.0, IBM Corp., Armonk, NY, USA). Means, standard deviations (SDs) and numbers were used to present the results of descriptive analyses. The normality of data was assessed by Kolmogorov-Smirnov test.

#### Factor analysis

The data were deemed appropriate for factor analysis if the Bartlett’s test of sphericity had a *p*-value less than 0.05 and the Kaiser-Meyer-Olkin measure of sampling adequacy exceeded 0.80 (Kaiser, 1974). An exploratory factor analysis was performed using maximum-likelihood extraction, or principal axis factoring if the data were not normally distributed (Costello & Osborne, 2005). Factors were extracted based on three criteria: the inflection point on the Scree plot (where factors above this point were retained), eigenvalues greater than 1.0, and each factor accounting for more than 10% of the variance (Costello & Osborne, 2005). Promax rotation with Kaiser normalization was utilized to estimate the loading of each item on the extracted factor. An item qualified for factor inclusion when it exhibited a factor loading coefficient greater than 0.3 (Costello & Osborne, 2005). McDonald’s omega total (ωt) was estimated to confirm that the factors derived from the factor analysis were reliable (Dunn, Baguley & Brunsden, 2014). An omega value above 0.70 is considered acceptable (McDonald, 2013).

#### Floor and ceiling effects

To determine whether there are any floor and ceiling effects, the distribution of the CSI-Ar score was examined. The lowest possible score on the CSI-Ar is (0 = floor), while the highest possible score is (100 = ceiling). Reliability and validity of a scale can be compromised if the percentage of participants with the lowest and highest scores exceeded 15% (Terwee et al., 2007).

#### Internal consistency and test-retest reliability

Cronbach’s alpha test has been utilized to assess the internal consistency of the CSI-Ar items. A value range of 0.70 to 0.95 for Cronbach’s alpha indicates acceptable internal consistency (Terwee et al., 2007). For analysis of test-retest reliability (consistency and stability of the CSI-Ar tool over time where no changes expected), a 2-week interval between two measures was required. A previous study recommended the test-retest time interval of 2 weeks which was long enough to prevent participants from forgetting previous answers but short enough to avoid changes in health conditions affecting replies (De Vet et al., 2011).

For the assessment of test-retest reliability of the CSI-Ar, the two-way random intraclass correlation coefficients (ICCs) for absolute agreement was used, along with the corresponding 95% confidence intervals (95% CIs) (Terwee et al., 2007). The reliability was rated as “excellent” (ICC ≥ 0.75), “good” (0.40 ≤ ICC < 0.75), or “poor” (ICC < 0.40). The standard error of measurement (SEM) was computed using the formula: SEM = SD_pooled standard deviation_ × √(1 − ICC). The smallest detectable change for the individual score (SDC_individual_) was computed using the following formula: SDC_individual_ = 1.96 × √2 × SEM. Then, the smallest detectable change for the group score (SDC_group_) was computed using the following formula: SDC_group_ = SDC_individual_/√n, where n means number of participants (Terwee et al., 2007).

#### Construct validity

To assess to which degree the CSI-Ar tool is assessing the concept it claims to measure, the construct validity was assessed. The Spearman’s rho correlation was used to assess the construct validity between total CSI-Ar scores and scores of the PCS, DASS-21, TSK, EQ-5D, and EuroQol VAS. The correlation was classified as “weak” (Spearman’s rho < 0.3), “medium” (0.3 ≤ Spearman’s rho < 0.5) or “strong” (Spearman’s rho ≥ 0.5) (Cohen, 2016).

#### Discriminant validity

To ensure that the CSI-Ar tool is measuring the specific concept it claims to measure and not overlapping with unrelated constructs, the discriminant validity was assessed. The Separate Mann-Whitney U-tests were performed to test the discriminant validity of the total CSI-Ar score between 1) patients’ group and healthy control group, 2) patients with at least one physician-diagnosed disorder in Part B of the CSI-Ar and patients without physician-diagnosed disorders, and 3) patients with self-reported single site pain and patients with self-reported multisite pain (pain in two or more sites).

## Results

The CSI was forward and backward translated into Arabic without any major obstacles. In the pre-testing phase, no comprehensibility issues emerged, as the participants reported that items of the CSI–Ar were clear and easy to understand. Therefore, no changes were made to the CSI–Ar version after the pretest phase. The results of the Kolmogorov-Smirnov tests indicated that none of the outcome measures followed a normal distribution (*P* < 0.01).

### Participants’ characteristics

Table 1 presents the demographic characteristics of participants with different musculoskeletal pain disorders (*n* = 264) and healthy controls (*n* = 56). The average scores of the CSI-Ar (Part A) were 31.59 ± 16.69 and 12.88 ± 12.51 for the patients and healthy controls, respectively. In Part B of the CSI-Ar, 76 patients (28.79%) reported at least one physician-diagnosed disorder, while the remaining patients did not report any diagnosis.

**Table 1:** Demographic and clinical characteristics of participants.

	Patients with different musculoskeletal pain disorders (*n* = 264)	Healthy controls (*n* = 56)
Age, year	42.66 ± 15.33	33.23 ± 15.86
Sex *n*		
Women	175	38
Men	89	18
Height, cm	161.44 ± 10.41	160.54 ± 10.11
Weight, kg	77.16 ± 17.84	63.91 ± 17.16
Education level, *n*		
Illiterate	24	0
Elementary	17	2
Middle	18	2
High	37	9
Bachelor’s degree	147	35
Master’s degree	18	8
PhD’s degree	3	0
Smoking status, *n*		
Current smoker	59	16
Former smoker	29	3
Never smoked	176	37
Marital status		
Married	139	19
Single	75	31
Divorced	32	6
Widowed	18	0
Number of pain sites, *n*		
0	6	–
1	143	–
2	53	–
3	31	–
4	12	–
5 or more	19	–
Most referred areas, *n*		
Neck	32	–
Shoulders	31	–
Upper back	23	–
Low back	35	–
Elbow	7	–
Wrists/hands	16	–
Hips/thighs	23	–
Knees	33	–
Ankles/feet	24	–
CSI-Ar, Part A	27.97 ± 16.08	11.27 ± 11.50
CSI-Ar, Part B, *n*		
Restless leg syndrome	6	0
Chronic fatigue syndrome	10	0
Fibromyalgia	4	0
Temporomandibular joint disorder	2	1
Migraine or tension headaches	23	5
Irritable bowel syndrome	45	9
Multiple chemical sensitivities	4	2
Neck injury (including whiplash)	5	0
Anxiety or panic attacks	18	1
Depression	21	3
NPRS	6.40 ± 2.26	–
Pain catastrophizing scale		
Rumination	6.48 ± 3.93	–
Magnification	3.67 ± 3.25	–
Helplessness	7.64 ± 6.15	–
Total score	17.79 ± 12.19	–
Depression, Anxiety and stress scale		–
Depression	5.33 ± 4.79	–
Anxiety	4.10 ± 4.43	–
Stress	6.69 ± 4.69	–
Total score	16.12 ± 12.72	–
Tampa scale of Kinesiophobia	37.17 ± 8.32	–
5-level EuroQol-5D	0.67 ± 0.27	–
EuroQol VAS	63.29 ± 29.43	–

**Notes:**

Data are presented as mean ± SD. Abbreviations: CSI-Ar, Arabic version of the Central Sensitization Inventory, NPRS, Numeric pain rating scale.

### Factor analysis

**Figure 1:**
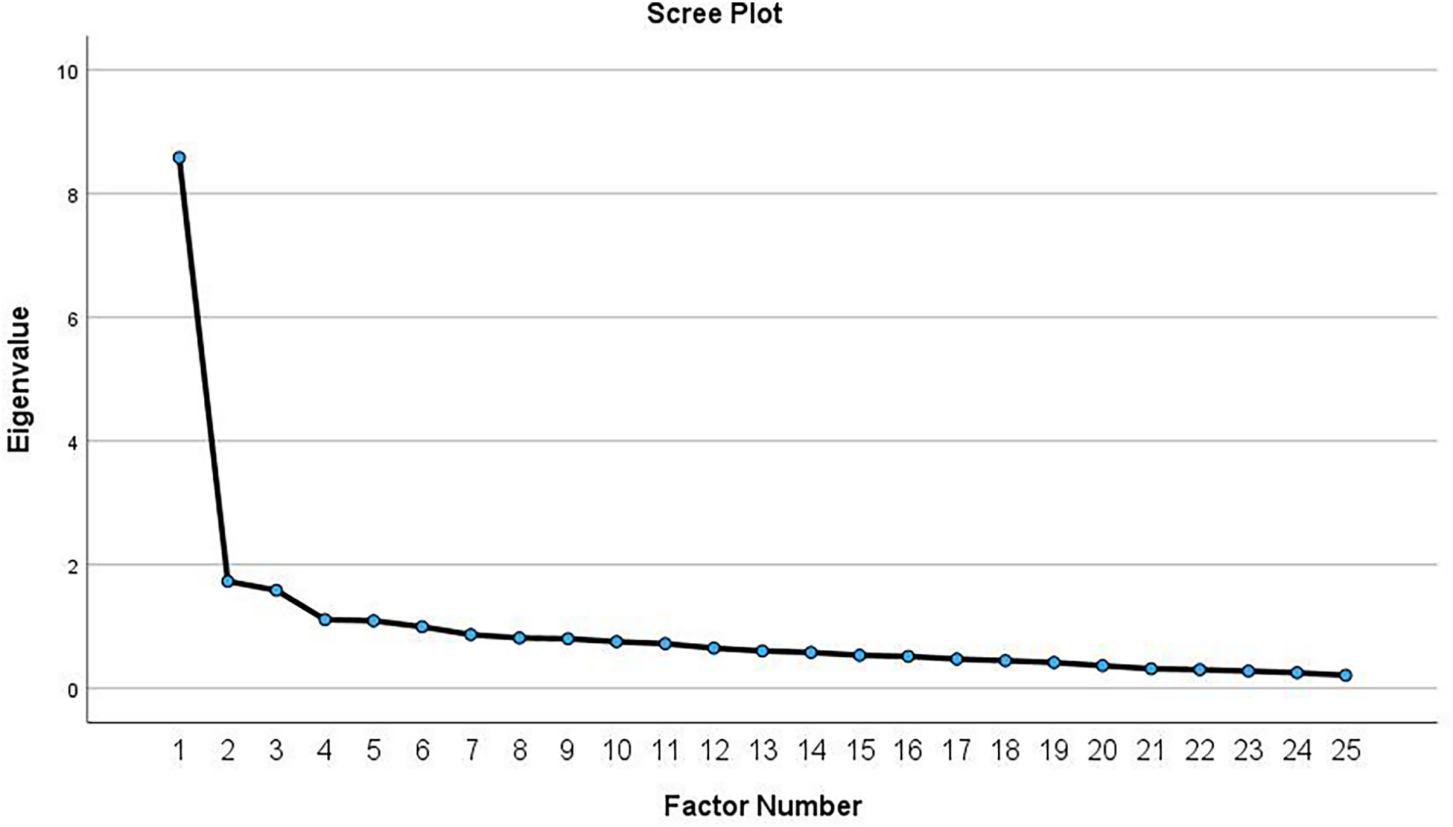
Scree plot from the exploratory factor analysis of the CSI-Ar among patients with chronic musculoskeletal pain.

The results of the KMO and Bartlett’s test indicate that the data are suitable for factor analysis, with a KMO of 0.915 and a significant Bartlett’s test of sphericity (*P* < 0.001). The principal axis factoring revealed five possible eigenvalues greater than 1.0. The first eigenvalue (8.58) accounted for 32.4% of the total variance, whereas the second to the fifth eigenvalues (ranged between 1.73 and 1.09) accounted for <10% of the variance. Visual inspection of the Scree plot (Fig. 1) showed inflection point at the second eigenvalue, which indicated a 1-factor model. After re-running the analysis to extract a single factor, this factor accounted for 31.9% of the total variance. As shown in Table 2, two items (items 10 and 18) were not loaded in the factor matrix. Removing these items resulted in a one-factor solution comprising 23 items, explaining 34.2% of the variance. The unidimensionality of the CSI-Ar (part A) was confirmed by the high value of McDonald’s omega total (ωt = 0.92). The subsequent measurement properties were conducted using this established factor structure. The total scores of the 23-item CSI-Ar ranges from 0–92.

**Table 2:** Factor loadings extracted using principal axis factor and promax rotation with Kaiser normalization.

Individual items	Factor 1
1. I feel tired and unrefreshed when I wake from sleeping.	0.619
2. My muscles feel stiff and achy.	0.416
3. I have anxiety attacks.	0.515
4. I grind or clench my teeth.	0.335
5. I have problems with diarrhea and/or constipation.	0.551
6. I need help in performing my daily activities.	0.597
7. I am sensitive to bright lights.	0.534
8. I get tired very easily when I am physically active.	0.66
9. I feel pain all over my body.	0.727
**10. I have headaches.**	
11. I feel discomfort in my bladder and/or burning when I urinate.	0.637
12. I do not sleep well.	0.555
13. I have difficulty concentrating.	0.745
14. I have skin problems such as dryness, itchiness, or rashes.	0.468
15. Stress makes my physical symptoms get worse.	0.665
16. I feel sad or depressed.	0.632
17. I have low energy.	0.782
**18. I have muscle tension in my neck and shoulders.**	
19. I have pain in my jaw.	0.321
20. Certain smells, such as perfumes, make me feel dizzy and nauseated.	0.439
21. I have to urinate frequently.	0.684
22. My legs feel uncomfortable and restless when I am trying to go to sleep at night.	0.662
23. I have difficulty remembering things.	0.678
24. I suffered trauma as a child.	0.365
25. I have pain in my pelvic area.	0.533

**Note:**

*Bolded items were not loaded on the factor matrix.

### Floor and ceiling effects

No floor and ceiling effects were found. None of the patients scored the lowest (sum score = 0) or highest (sum score = 100) score on the CSI-Ar scale. Of the 264 patients, only one patient (0.4%) had the lowest CSI-Ar score, whereas none of the patients had the highest CSI-Ar score.

### Internal consistency

The internal consistency of the CSI-Ar was considered to be acceptable whereas the Cronbach’s alpha value was 0.919.

### Test-retest reliability

The CSI-Ar was completed twice by 47 patients, with a 2-week delay between the two assessments. The CSI-Ar’s test-retest reliability was excellent, with an ICC_2, 1_ of 0.874 (95% CI [0.785–0.928], *P* < 0.001). The SEM was 5.80, the SDC_individual_ was 16.09 and the SDC_group_ was 2.23. The average scores of the CSI-Ar were 27.64 ± 16.58 at the first test and 27.23 ± 16.14 at the second test. The Wilcoxon Signed Rank test showed no significant differences between the first and second average scores of the CSI-Ar (*P* = 0.86).

### Construct validity

The results showed that the CSI-Ar scale had significant correlations with all PCS subscales and total score (Spearman’s rho = 0.459–0.563, *P* < 0.001), all DASS-21 subscales and total score (Spearman’s rho = 0.599–0.685, *P* < 0.001), the TSK (Spearman’s rho = 0.395, *P* < 0.001), and the EQ-5D (Spearman’s rho = −0.396, *P* < 0.001). The results of the correlation analyses are demonstrated in detail in Table 3.

**Table 3:** Correlations between the CSI-Ar scale and other outcome measures in patients with different musculoskeletal pain disorders (*n* = 264).

	CSI-Ar scale	
	Spearman’s rho	*P*
NPRS	0.282	<0.001
Pain catastrophizing scale		
Rumination	0.469	<0.001
Magnification	0.459	<0.001
Helplessness	0.563	<0.001
Total score	0.552	<0.001
Depression, Anxiety and Stress Scale (DASS-21)		
Depression	0.625	<0.001
Anxiety	0.655	<0.001
Stress	0.599	<0.001
Total score	0.685	<0.001
Tampa Scale of Kinesiophobia	0.395	<0.001
5-level EuroQol-5D	−0.396	<0.001
EuroQol VAS	−0.222	<0.001

**Note:**

Abbreviations: CSI-Ar, Arabic version of the Central Sensitization Inventory; NPRS, numeric pain rating scale; VAS, Visual analog scale.

### Discriminative validity

The Mann-Whitney U-test showed a statistically significant difference between the patient group and the healthy control group (*P* < 0.001), with the healthy controls displaying a lower average CSI-Ar score (12.27 ± 11.50) when compared to the patient group (27.97 ± 16.08) (Table 1).

When compared to patients with at least one physician-diagnosed disorder in Part B of the CSI-Ar (*n* = 76), the Mann-Whitney U-test revealed that patients without physician-diagnosed disorders (*n* = 188) had a significantly lower average CSI-Ar score (36.97 ± 17.11 *vs*. 24.33 ± 14.14, respectively, *P* < 0.001). Further, the patients with self-reported single-site pain (*n* = 143) had a statistically significantly lower CSI-Ar score when compared to patients with self-reported multisite pain (*n* = 115) (25.04 ± 15.23 *vs*. 31.29 ± 15.99, *P* = 0.001).

## Discussion

This study translated, culturally adapted, and validated the CSI-Ar version in patients with different chronic musculoskeletal pain disorders. The results showed that the CSI-Ar has satisfactory psychometric properties, when compared to those of the original English version (Mayer et al., 2012).

The structural validity of the CSI has shown variability across different languages (Pitance et al., 2016; Cuesta-Vargas et al., 2016; Tanaka et al., 2017; Chiarotto et al., 2018; Knezevic et al., 2018; van der Noord, Paap & van Wilgen, 2018). However, a recent multi-national study suggested that only total CSI scores should be used and reported (Cuesta-Vargas et al., 2018), confirming its unidimensionality and validating the use of the total score. Our research aligns with this study, supporting the unidimensionality of the CSI-Ar. Our factor analysis revealed a one-factor solution that accounted for a significant proportion of variance, providing evidence of structural validity. The results of the current study are similar to other adaption studies such as Italian (Chiarotto et al., 2018) and Spanish (Cuesta-Vargas et al., 2016). However, these findings differ from the English (Mayer et al., 2012), Dutch (Kregel et al., 2016), and Brazilian Portuguese (Caumo et al., 2017) versions, which identified more than one factor.

The internal consistency of the CSI-Ar was considered to be acceptable with a Cronbach’s alpha value of 0.915. This finding has been found to be consistent with the values of the internal consistency of the other versions of the CSI scale of other languages which had Cronbach’s alpha values ranging from 0.87 (Chiarotto et al., 2018; Sharma et al., 2020) to 0.993 (Bilika et al., 2020). Furthermore, the CSI-Ar’s test-retest reliability was excellent, with an ICC of 0.872, which is comparable to the results of previous studies that had test-retest reliability values ranging from 0.85 (Tanaka et al., 2017a) to 0.991(Bilika et al., 2020). The SEM in our study was 6.19 and the SDC was 2.38, whereas the SEM values of other studies ranged from 0.31 (Sharma et al., 2020) to 3.16 (Knezevic et al., 2018), and the SDC ranged from 0.86 (Sharma et al., 2020) to 8.12 (Knezevic et al., 2018). One possible explanation for the higher SEM in our study could be related to the characteristics of our study sample. It is important to consider the demographics and clinical characteristics of the participants in our study, as these factors can influence the variability of responses and ultimately impact the SEM. Additionally, differences in the administration of the CSI-Ar, such as variations in the instructions given to participants or the setting in which the assessment took place, could contribute to the variation in SEM values across studies.

Assessing construct validity, the results showed that the CSI-Ar scale had positive medium to strong correlations with all PCS subscales. Previous versions of the scale of the other languages reported also a significant correlation between the PCS subscales and CSI (Caumo et al., 2017; Bilika et al., 2020). Moreover, the CSI-Ar scale showed a positive and strong correlation with the DASS-21 subscales and the total score, which is not in line with the German CSI version (Klute et al., 2021), as they reported that all the three scales of the DASS-21 showed a low, positive correlation with the German CSI version, indicating an increasing negative emotional stress with increasing scores. This discrepancy between the Arabic and German versions of the CSI suggests that the relationship between the CSI and DASS-21 scales may be influenced by cultural and contextual factors. Further research is needed to understand these differences and their implications for the use of the CSI in different cultural settings.

The results showed a significant difference between the patient group and the healthy control group, with the healthy controls displaying a lower average CSI-Ar score compared to the healthy control group. Moreover, when compared to patients with at least one physician-diagnosed disorder in Part B of the CSI-Ar, the results revealed that patients without physician-diagnosed disorders had a significantly lower average CSI-Ar score. Furthermore, patients with self-reported single-site pain had a significantly lower CSI-Ar score when compared to patients with self-reported multisite pain. The results were expected since each of these groups had a different level of CS.

One limitation of the current study is that Saudi Arabia is a multicultural country, and its residents speak different accents, which may influence some concepts and aspects of the CSI-Ar. It is recommended to conduct further validation studies of the CSI-Ar in larger and more diverse populations, including individuals from different regions and cultural backgrounds within Saudi Arabia, to account for potential linguistic and cultural variations. Another limitation is that women outnumbered men in this study, which may affect the concepts of pain and, consequently, the study results. It is recommended to conduct further studies investigating the influence of gender differences on the interpretation and response patterns to the CSI-Ar.

## Conclusion

The CSI-Ar was found to be internally consistent, reliable, and valid in Arabic-speaking participants who complained of chronic musculoskeletal pain. The results showed excellent test-retest reliability and acceptable internal consistency. Moreover, the results had found that the psychometric properties of the CSI-Ar corresponded to the original English version and the other previous CSI versions of various languages. The CSI-Ar can be a useful screening tool for the clinicians and researchers to assess the CS/CSS-related symptoms in participants with chronic musculoskeletal pain. Future research should explore the responsiveness of the CSI-Ar in detecting changes in central sensitization over time or in response to treatment, to establish its utility for monitoring and guiding interventions. Additionally, it is recommended to assess the predictive validity of the CSI-Ar in identifying subgroups of patients who may benefit from targeted interventions for chronic musculoskeletal pain.

## Supplemental Information

10.7717/peerj.18251/supp-1 Supplemental Information 1 Central Sensitization Inventory (CSI): Arabic translation.

10.7717/peerj.18251/supp-2 Supplemental Information 2 Dataset.

## Additional Information and Declarations

### Data Availability

The following information was supplied regarding data availability: The raw measurements are available in the Supplemental File.

## References

[ref-1] Aaron LA, Buchwald D (2001). A review of the evidence for overlap among unexplained clinical conditions. Annals of Internal Medicine.

[ref-2] Alghadir AH, Anwer S, Iqbal ZA (2016). The psychometric properties of an Arabic numeric pain rating scale for measuring osteoarthritis knee pain. Disability and Rehabilitation.

[ref-3] Ali AM, Ahmed A, Sharaf A, Kawakami N, Abdeldayem SM, Green J (2017). The Arabic version of the depression anxiety stress scale-21: cumulative scaling and discriminant-validation testing. Asian Journal of Psychiatry.

[ref-4] Beaton DE, Bombardier C, Guillemin F, Ferraz MB (2000). Guidelines for the process of cross-cultural adaptation of self-report measures. Spine.

[ref-5] Bekairy AM, Bustami RT, Almotairi M, Jarab A, Katheri AM, Aldebasi TM, Aburuz S (2018). Validity and reliability of the Arabic version of the the EuroQOL (EQ-5D). A study from Saudi Arabia. International Journal of Health Sciences (Qassim).

[ref-6] Bilika P, Neblett R, Georgoudis G, Dimitriadis Z, Fandridis E, Strimpakos N, Kapreli E (2020). Cross-cultural adaptation and psychometric properties of the greek version of the central sensitization inventory. Pain Practice.

[ref-7] Boateng GO, Neilands TB, Frongillo EA, Melgar-Quiñonez HR, Young SL (2018). Best practices for developing and validating scales for health, social, and behavioral research: a primer. Frontiers in Public Health.

[ref-8] Caumo W, Antunes LC, Elkfury JL, Herbstrith EG, Sipmann RB, Souza A, Torres ILS, Dos Santos VS, Neblett R (2017). The central sensitization inventory validated and adapted for a Brazilian population: psychometric properties and its relationship with brain-derived neurotrophic factor. Journal of Pain Research.

[ref-9] Chiarotto A, Viti C, Sulli A, Cutolo M, Testa M, Piscitelli D (2018). Cross-cultural adaptation and validity of the Italian version of the central sensitization inventory. Musculoskeletal Science and Practice.

[ref-10] Cieza A, Causey K, Kamenov K, Hanson SW, Chatterji S, Vos T (2020). Global estimates of the need for rehabilitation based on the global burden of disease study 2019: a systematic analysis for the global burden of disease study 2019. The Lancet.

[ref-11] Cimmino MA, Ferrone C, Cutolo M (2011). Epidemiology of chronic musculoskeletal pain. Best Practice and Research: Clinical Rheumatology.

[ref-12] Cohen J (2016). A power primer. Psychological Bulletin.

[ref-13] Costello AB, Osborne J (2005). Best practices in exploratory factor analysis: four recommendations for getting the most from your analysis. Practical Assessment, Research, and Evaluation.

[ref-14] Cuesta-Vargas AI, Neblett R, Chiarotto A, Kregel J, Nijs J, van Wilgen CP, Pitance L, Knezevic A, Gatchel RJ, Mayer TG (2018). Dimensionality and reliability of the central sensitization inventory in a pooled multicountry sample. The Journal of Pain.

[ref-15] Cuesta-Vargas AI, Roldan-Jimenez C, Neblett R, Gatchel RJ (2016). Cross-cultural adaptation and validity of the Spanish central sensitization inventory. SpringerPlus.

[ref-16] Curatolo M, Petersen-Felix S, Arendt-Nielsen L, Giani C, Zbinden AM, Radanov BP (2001). Central hypersensitivity in chronic pain after whiplash injury. The Clinical Journal of Pain.

[ref-17] De Vet HCW, Terwee CB, Mokkink LB, Knol DL (2011). Measurement in medicine: a practical guide. Measurement in Medicine: A Practical Guide.

[ref-18] Downie WW, Leatham PA, Rhind VM, Wright V, Branco JA, Anderson JA (1978). Studies with pain rating scales. Annals of the Rheumatic Diseases.

[ref-19] Dunn TJ, Baguley T, Brunsden V (2014). From alpha to omega: a practical solution to the pervasive problem of internal consistency estimation. British Journal of Psychology.

[ref-20] Gagnier JJ, Lai J, Mokkink LB, Terwee CB (2021). COSMIN reporting guideline for studies on measurement properties of patient-reported outcome measures. Quality of Life Research.

[ref-21] Jensen MP, McFarland CA (1993). Increasing the reliability and validity of pain intensity measurement in chronic pain patients. Pain.

[ref-22] Jensen MP, Turner JA, Romano JM (1994). What is the maximum number of levels needed in pain intensity measurement?. Pain.

[ref-23] Jensen MP, Turner JA, Romano JM, Fisher LD (1999). Comparative reliability and validity of chronic pain intensity measures. Pain.

[ref-24] Jiang LC, Yan YJ, Jin ZS, Hu ML, Wang L, Song Y, Li NN, Su J, Wu DX, Xiao T (2020). The depression anxiety stress scale-21 in chinese hospital workers: reliability, latent structure, and measurement invariance across genders. Frontiers in Psychology.

[ref-25] Jull G, Sterling M, Kenardy J, Beller E (2007). Does the presence of sensory hypersensitivity influence outcomes of physical rehabilitation for chronic whiplash?—a preliminary RCT. Pain.

[ref-26] Kaiser HF (1974). An index of factorial simplicity. Psychometrika.

[ref-27] Katz J, Melzack R (1999). Measurement of pain. Surgical Clinics of North America.

[ref-28] Klute M, Laekeman M, Kuss K, Petzke F, Dieterich A, Leha A, Neblett R, Ehrhardt S, Ulma J, Schäfer A (2021). Cross-cultural adaptation and validation of the German Central Sensitization Inventory (CSI-GE). BMC Musculoskeletal Disorders.

[ref-29] Knezevic A, Neblett R, Jeremic-Knezevic M, Tomasevic-Todorovic S, Boskovic K, Colovic P, Cuesta-Vargas A (2018). Cross-cultural adaptation and psychometric validation of the serbian version of the central sensitization inventory. Pain Practice.

[ref-30] Kregel J, Vuijk PJ, Descheemaeker F, Keizer D, Van Der Noord R, Nijs J, Cagnie B, Meeus M, Van Wilgen P (2016). The dutch Central Sensitization Inventory (CSI). Clinical Journal of Pain.

[ref-31] Latremoliere A, Woolf CJ (2009). Central sensitization: a generator of pain hypersensitivity by central neural plasticity. Journal of Pain.

[ref-32] Lluch Girbés E, Nijs J, Torres-Cueco R, López Cubas C (2013). Pain treatment for patients with osteoarthritis and central sensitization. Physical Therapy.

[ref-33] Lovibond SH (1995). Manual for the depression anxiety stress scales. Sydney Psychology Foundation.

[ref-34] Madi M, Hamzeh H, Abujaber S, Altubasi I (2022). Cross cultural adaptation, validity, and reliability of central sensitization inventory in Arabic language. Disability and Rehabilitation.

[ref-35] Mayer TG, Neblett R, Cohen H, Howard KJ, Choi YH, Williams MJ, Perez Y, Gatchel RJ (2012). The development and psychometric validation of the central sensitization inventory. Pain Practice.

[ref-36] McDonald RP (2013). Test theory: a unified treatment.

[ref-37] Meyer K, Sprott H, Mannion AF (2008). Cross-cultural adaptation, reliability, and validity of the German version of the pain catastrophizing scale. Journal of Psychosomatic Research.

[ref-38] Miller RP, Kori SH, Todd DD (1991). The Tampa Scale: a measure of kinisophobia. The Clinical Journal of Pain.

[ref-39] Neblett R (2018). The central sensitization inventory: a user’s manual. Journal of Applied Biobehavioral Research.

[ref-40] Nijs J, Torres-Cueco R, Van Wilgen P, Lluch Girbés E, Struyf F, Roussel N, Van Oosterwijck J, Daenen L, Kuppens K, Vanderweeen L (2014). Applying modern pain neuroscience in clinical practice: criteria for the classification of central sensitization pain. Pain Physician.

[ref-41] Pezirkianidis C, Karakasidou E, Lakioti A, Stalikas A, Galanakis M (2018). Psychometric Properties of the depression, anxiety, stress scales-21 (DASS-21) in a greek sample. Psychology.

[ref-42] Pitance L, Piraux E, Lannoy B, Meeus M, Berquin A, Eeckhout C, Dethier V, Robertson J, Meeus M, Roussel N (2016). Cross cultural adaptation, reliability and validity of the French version of the central sensitization inventory. Manual Therapy.

[ref-43] Price DD, Bush FM, Long S, Harkins SW (1994). A comparison of pain measurement characteristics of mechanical visual analogue and simple numerical rating scales. Pain.

[ref-44] Rabin R, De Charro F (2001). EQ-5D: a measure of health status from the EuroQol Group. Annals of Medicine.

[ref-45] Roussel NA, Nijs J, Meeus M, Mylius V, Fayt C, Oostendorp R (2013). Central sensitization and altered central pain processing in chronic low back pain fact or myth?. The Clinical Journal of Pain.

[ref-46] Sharma S, Jha J, Pathak A, Neblett R (2020). Translation, cross-cultural adaptation, and measurement properties of the Nepali version of the Central Sensitization Inventory (CSI). BMC Neurology.

[ref-47] Sullivan MJL, Bishop SR, Pivik J (1995). The pain catastrophizing scale: development and validation. Psychological Assessment.

[ref-48] Tanaka K, Nishigami T, Mibu A, Manfuku M, Yono S, Shinohara Y, Tanabe A, Ono R (2017). Validation of the Japanese version of the central sensitization inventory in patients with musculoskeletal disorders. PLOS ONE.

[ref-49] Terkawi AS, Sullivan M, Abolkhair A, Al-Zhahrani T, Terkawi RS, Alasfar EM, Khait SSA, Elkabbani A, Kabbani N, Altirkawi KA, Tsang S (2017). Development and validation of Arabic version of the pain catastrophizing scale. Saudi Journal of Anaesthesia.

[ref-50] Terwee CB, Bot SDM, de Boer MR, van der Windt DAWM, Knol DL, Dekker J, Bouter LM, de Vet HCW (2007). Quality criteria were proposed for measurement properties of health status questionnaires. Journal of Clinical Epidemiology.

[ref-51] van der Noord R, Paap D, van Wilgen CP (2018). Convergent validity and clinically relevant categories for the Dutch central sensitization inventory in patients with chronic pain. Journal of Applied Biobehavioral Research.

[ref-52] Vierck CJ (2006). Mechanisms underlying development of spatially distributed chronic pain (fibromyalgia). Pain.

[ref-53] Von Elm E, Altman DG, Egger M, Pocock SJ, Gøtzsche PC, Vandenbroucke JP (2007). The strengthening the reporting of observational studies in epidemiology (STROBE) statement: guidelines for reporting observational studies. The Lancet.

[ref-54] Vos T, Abajobir AA, Abate KH, Abbafati C, Abbas KM, Abd-Allah F, Abdulkader RS, Abdulle AM, Abebo TA, Abera SF, Aboyans V, Abu-Raddad LJ, Ackerman IN, Adamu AA, Adetokunboh O, Afarideh M, Afshin A, Agarwal SK, Aggarwal R, Agrawal A, Agrawal S, Ahmadieh H, Ahmed MB, Aichour MTE, Aichour AN, Aichour I, Aiyar S, Akinyemi RO, Akseer N, Al Lami FH, Alahdab F, Al-Aly Z, Alam K, Alam N, Alam T, Alasfoor D, Alene KA, Ali R, Alizadeh-Navaei R, Alkerwi A, Alla F, Allebeck P, Allen C, Al-Maskari F, Al-Raddadi R, Alsharif U, Alsowaidi S, Altirkawi KA, Amare AT, Amini E, Ammar W, Amoako YA, Andersen HH, Antonio CAT, Anwari P, Ärnlöv J, Artaman A, Aryal KK, Asayesh H, Asgedom SW, Assadi R, Atey TM, Atnafu NT, Atre SR, Avila-Burgos L, Avokphako EFGA, Awasthi A, Bacha U, Badawi A, Balakrishnan K, Banerjee A, Bannick MS, Barac A, Barber RM, Barker-Collo SL, Bärnighausen T, Barquera S, Barregard L, Barrero LH, Basu S, Battista B, Battle KE, Baune BT, Bazargan-Hejazi S, Beardsley J, Bedi N, Beghi E, Béjot Y, Bekele BB, Bell ML, Bennett DA, Bensenor IM, Benson J, Berhane A, Berhe DF, Bernabé E, Betsu BD, Beuran M, Beyene AS, Bhala N, Bhansali A, Bhatt S, Bhutta ZA, Biadgilign S, Bicer BK, Bienhoff K, Bikbov B, Birungi C, Biryukov S, Bisanzio D, Bizuayehu HM, Boneya DJ, Boufous S, Bourne RRA, Brazinova A, Brugha TS, Buchbinder R, Bulto LNB, Bumgarner BR, Butt ZA, Cahuana-Hurtado L, Cameron E, Car M, Carabin H, Carapetis JR, Cárdenas R, Carpenter DO, Carrero JJ, Carter A, Carvalho F, Casey DC, Caso V, Castañeda-Orjuela CA, Castle CD, Catalá-López F, Chang HY, Chang JC, Charlson FJ, Chen H, Chibalabala M, Chibueze CE, Chisumpa VH, Chitheer AA, Christopher DJ, Ciobanu LG, Cirillo M, Colombara D, Cooper C, Cortesi PA, Criqui MH, Crump JA, Dadi AF, Dalal K, Dandona L, Dandona R, das Neves J, Davitoiu DV, de Courten B, De DLD, Defo BK, Degenhardt L, Deiparine S, Dellavalle RP, Deribe K, Des Jarlais DC, Dey S, Dharmaratne SD, Dhillon PK, Dicker D, Ding EL, Djalalinia S, Do HP, Dorsey ER, dos Santos KPB, Douwes-Schultz D, Doyle KE, Driscoll TR, Dubey M, Duncan BB, El-Khatib ZZ, Ellerstrand J, Enayati A, Endries AY, Ermakov SP, Erskine HE, Eshrati B, Eskandarieh S, Esteghamati A, Estep K, Fanuel FBB, Farinha CSES, Faro A, Farzadfar F, Fazeli MS, Feigin VL, Fereshtehnejad SM, Fernandes JC, Ferrari AJ, Feyissa TR, Filip I, et al (2017). Global, regional, and national incidence, prevalence, and years lived with disability for 328 diseases and injuries for 195 countries, 1990–2016: a systematic analysis for the Global Burden of Disease study 2016. The Lancet.

[ref-55] Wijma AJ, van Wilgen CP, Meeus M, Nijs J (2016). Clinical biopsychosocial physiotherapy assessment of patients with chronic pain: the first step in pain neuroscience education. Physiotherapy Theory and Practice.

[ref-56] Woolf CJ (2011). Central sensitization: implications for the diagnosis and treatment of pain. Pain.

[ref-57] Yangui N, Yahia A, Ghroubi S, Elleuch MH (2017). Translation and validation of the Tampa Scale of Kinesiophobia Arabic version in chronic low back pain. Annals of Physical and Rehabilitation Medicine.

[ref-58] Yıldırım A, Boysan M, Kefeli MC (2018). Psychometric properties of the Turkish version of the Depression Anxiety Stress Scale-21 (DASS-21). British Journal of Guidance and Counselling.

[ref-59] Yunus MB (2007). Fibromyalgia and overlapping disorders: the unifying concept of central sensitivity syndromes. Seminars in Arthritis and Rheumatism.

[ref-60] Zanon C, Brenner RE, Baptista MN, Vogel DL, Rubin M, Al-Darmaki FR, Gonçalves M, Heath PJ, Liao HY, Mackenzie CS, Topkaya N, Wade NG, Zlati A (2021). Examining the dimensionality, reliability, and invariance of the depression, anxiety, and stress scale-21 (DASS-21) across eight countries. Assessment.

