# Supplementary material for: Cross-cultural adaptation and psychometric properties of the Arabic version of the Central Sensitization Inventory in people with chronic musculoskeletal pain
Source: PeerJ. 2024 Oct 8;12:e18251. doi: 10.7717/peerj.18251 (PMC11468962; doi:10.7717/peerj.18251)
Supplement: Supplemental Information 1 [file peerj-12-18251-s001.pdf]

قائمة جرد الحساسية المركزية (الجزء أ)

الاسم: **التاريخ:**

يرجى وضع دائرة حول أفضل إجابة أمام كل عباره:

١	أشعر بالتعب وعدم الانتعاش عندما أستيقظ من النوم	ابدا	نادرا	أحيانا	غالبا	دائما
٢	تشعر عضلاتي بالتيبس والألم	ابدا	نادرا	أحيانا	غالبا	دائما
٣	لدي نوبات قلق	ابدا	نادرا	أحيانا	غالبا	دائما
٤	أنا أطحن أسناني	ابدا	نادرا	أحيانا	غالبا	دائما
٥	لدي مشاكل مع الإسهال و / أو الإمساك	ابدا	نادرا	أحيانا	غالبا	دائما
٦	أحتاج إلى مساعدة في أداء أنشطتي اليومية	ابدا	نادرا	أحيانا	غالبا	دائما
٧	أنا حساس للأضواء الساطعة	ابدا	نادرا	أحيانا	غالبا	دائما
٨	أشعر بالتعب بسهولة شديدة عندما أكون نشيطًا بدنيًا	ابدا	نادرا	أحيانا	غالبا	دائما
٩	أشعر بالألم في جميع أنحاء جسدي	ابدا	نادرا	أحيانا	غالبا	دائما
١٠	انا أعاني من الصداع	ابدا	نادرا	أحيانا	غالبا	دائما
١١	أشعر بعدم الراحة في مثانتي و / أو حرقان عند التبول	ابدا	نادرا	أحيانا	غالبا	دائما
١٢	انا لا أنام جيداً	ابدا	نادرا	أحيانا	غالبا	دائما
١٣	أجد صعوبة في التركيز	ابدا	نادرا	أحيانا	غالبا	دائما
١٤	أعاني من مشاكل جلدية مثل الجفاف أو الحكة أو الطفح الجلدي	ابدا	نادرا	أحيانا	غالبا	دائما
١٥	الإجهاد يجعل أعراضي الجسدية تزداد سوءًا	ابدا	نادرا	أحيانا	غالبا	دائما
١٦	أشعر بالحزن أو الاكتئاب	ابدا	نادرا	أحيانا	غالبا	دائما
١٧	لدي طاقة منخفضة	ابدا	نادرا	أحيانا	غالبا	دائما
١٨	لدي توتر عضلي في رقبتي وكتفي	ابدا	نادرا	أحيانا	غالبا	دائما
١٩	لدي ألم في فكي	ابدا	نادرا	أحيانا	غالبا	دائما
٢٠	بعض الروائح ، مثل العطور ، تجعلني أشعر بالدوار والغثيان	ابدا	نادرا	أحيانا	غالبا	دائما
٢١	لا بد لي من التبول بشكل متكرر	ابدا	نادرا	أحيانا	غالبا	دائما
٢٢	أشعر بعدم الارتياح في ساقيّ وضيق عندما أحاول النوم ليلاً	ابدا	نادرا	أحيانا	غالبا	دائما
٢٣	أجد صعوبة في تذكر الأشياء	ابدا	نادرا	أحيانا	غالبا	دائما
٢٤	لقد عانيت من صدمة عندما كنت طفلة	ابدا	نادرا	أحيانا	غالبا	دائما
٢٥	أعاني من ألم في منطقة الحوض	ابدا	نادرا	أحيانا	غالبا	دائما
المجموع =						

قائمة جرد الحساسية المركزية (الجزء ب)

هل تم تشخيصك من قبل الطبيب بأي من الاضطرابات التالية؟
يرجى تحديد المربع الموجود أمام كل تشخيص وكتابة سنة التشخيص.

		نعم	لا	سنة التشخيص
١	متلازمة تململ الساق			
٢	متلازمة التعب المزمن			
٣	متلازمة الألم العضلي الليفي			
٤	اضطراب المفصل الصدغي الفكي			
٥	الصداع النصفي أو صداع التوتر			
٦	متلازمة القولون العصبي			
٧	الحساسيات الكيميائية المتعددة			
٨	إصابة الرقبة (بما في ذلك إصابة الرقبة في حادث سير)			
٩	القلق أو نوبات الهلع			
١٠	الاكتئاب (كآبة)			
